# Supplementary material for: Interpretation of exercise-induced changes in human skeletal muscle mRNA expression depends on the timing of the post-exercise biopsies
Source: PeerJ. 2022 Feb 4;10:e12856. doi: 10.7717/peerj.12856 (PMC8820226; doi:10.7717/peerj.12856)
Supplement: Supplemental Information 1 — The time-point with the maximal fold changes, the geometric mean for maximal fold change with geometric standard deviation (GSD), the 95% Confidence Interval (CI), the p value determined by the Mann-Whitney test, the Xiao value determined by a novel posteriori information fusion scheme (Deshmukh et al., 2021; Xiao et al., 2014), the q value determined by a Benjamini-Hochberg false discovery rate (FDR) of <5%, and the adjusted p value (Adj p value) determined by one-way ANOVA with Dunnett test, are reported for each target gene. [file peerj-10-12856-s001.docx]

**Table S1** Summary of changes in mRNA content following a single session of High-Intensity Interval Exercise (HIIE), measured in 9 participants. The time-point with the maximal fold changes, the geometric mean for maximal fold change with geometric standard deviation (GSD), the 95% Confidence Interval (CI), the *p* value determined by the Mann-Whitney test, the Xiao value determined by a novel posteriori information fusion scheme (Deshmukh et al. 2021; Xiao et al. 2014), the *q* value determined by a Benjamini-Hochberg false discovery rate (FDR) of < 5%, and the adjusted *p value (*Adj *p* value*)* determined by one-way ANOVA with Dunnett test, are reported for each target gene.

| **Gene name** | **Time-point with highest or lowest fold change** | | **Maximal fold change relative to baseline; Geometric mean (GSD)** | | **95% CI for fold change** | ***p* value** | **Xiao value** | ***q* value** | **Adj *p* value** |
| --- | --- | --- | --- | --- | --- | --- | --- | --- | --- |
| *HSP1A1* | | 9 h | 2.9 (3.5) | 1.1 - 7.7 | | 0.1615 | 0.0592 | 0.3012 | 0.1488 |
| *SDHB* | | 9 h | 1.6 (1.8) | 1.1 - 2.5 | | 0.1359 | 0.2575 | 0.2758 | 0.3362 |
| *COX4-1* | | 9 h | 1.9 (2.1) | 1.1 - 3.5 | | 0.1359 | 0.1484 | 0.2758 | 0.4058 |
| *NDUFB3* | | 9 h | 2.1 (2.5) | 1.0 - 4.2 | | 0.2224 | 0.2067 | 0.3654 | 0.2411 |
| *VEGFA* | | 9 h | 1.3 (1.7) | 0.8 - 1.9 | | 0.0625 | 0.4017 | 0.1906 | 0.9997 |
| *PGC-1β* | | 9 h | 1.0 (2.8) | 0.5 - 2.2 | | 0.3765 | 0.9911 | 0.5248 | 0.8453 |
| *CS* | | 24 h | 1.7 (1.7) | 1.1 - 2.5 | | 0.0770 | 0.1557 | 0.2125 | 0.1962 |
| *TFAM* | | 24 h | 1.3 (1.4) | 1.0 - 1.6 | | 0.1615 | 0.5066 | 0.3012 | 0.1337 |
| *UQCRC2* | | 24 h | 1.4 (1.9) | 0.9 - 2.3 | | 0.1359 | 0.3725 | 0.2758 | 0.3368 |
| *PPARβ/δ* | | 24 h | 1.1 (1.6) | 1.1 - 3.3 | | 0.6665 | 0.9305 | 0.7729 | 0.8974 |
| *MFN2* | | 72 h | 1.5 (2.1) | 0.6 - 3.9 | | 0.2222 | 0.5333 | 0.3654 | 0.7458 |
