# Supplementary material for: Interpretation of exercise-induced changes in human skeletal muscle mRNA expression depends on the timing of the post-exercise biopsies
Source: PeerJ. 2022 Feb 4;10:e12856. doi: 10.7717/peerj.12856 (PMC8820226; doi:10.7717/peerj.12856)
Supplement: Supplemental Information 2 [file peerj-10-12856-s002.docx]

S2 Table: Raw Cq value for all target mRNA expression.

| Gene | Participant | Cq Value | | | | | | |
| --- | --- | --- | --- | --- | --- | --- | --- | --- |
|  |  | baseline | 0 h | 3 h | 9 h | 24 h | 48 h | 72 h |
| PGC-1α | A | 10.17 | 7.98 | 28.05 | 12.62 | 4.79 | 2.62 | 4.12 |
|  | B | 6.04 | 7.53 | 16.80 | 9.73 | 3.26 | 1.70 | 5.21 |
|  | C | 4.65 | 3.54 | 32.02 | 12.59 | 7.07 | 3.39 | 2.14 |
|  | D | 4.02 | 5.04 | 18.72 | 9.30 | 5.06 | 5.44 |  |
|  | E | 3.61 | 3.52 | 14.89 | 3.08 | 4.86 | 3.08 | 0.76 |
|  | F | 6.92 | 5.57 | 6.20 | 1.22 | 1.23 | 2.31 |  |
|  | G | 3.76 | 3.79 | 18.79 | 5.40 | 0.42 | 5.45 | 2.88 |
|  | H | 3.35 | 1.75 | 9.84 | 2.70 | 2.37 | 3.15 |  |
|  | I | 7.54 | 3.09 | 19.05 | 7.65 | 3.43 | 3.55 | 1.68 |
| PGC-1α4 | A | 0.27 | 0.19 | 0.84 | 0.45 | 0.12 | 0.08 | 0.15 |
|  | B | 0.17 | 0.19 | 1.07 | 0.47 | 0.12 | 0.08 | 0.14 |
|  | C | 0.15 | 0.13 | 2.61 | 0.42 | 0.20 | 0.13 | 0.08 |
|  | D | 0.16 | 0.14 | 0.81 | 0.38 | 0.16 | 0.19 |  |
|  | E | 0.10 | 0.12 | 0.76 | 0.15 | 0.19 | 0.07 | 0.03 |
|  | F | 0.20 | 0.25 | 0.19 | 0.06 | 0.06 | 0.10 |  |
|  | G | 0.18 | 0.11 | 1.34 | 0.24 | 0.02 | 0.20 | 0.15 |
|  | H | 0.22 | 0.09 | 0.46 | 0.19 | 0.13 | 0.16 |  |
|  | I | 0.34 | 0.15 | 1.21 | 0.48 | 0.09 | 0.18 | 0.10 |
| PPARα | A | 0.32 | 0.31 | 1.47 | 0.34 | 0.25 | 0.15 | 0.27 |
|  | B | 0.33 | 0.39 | 1.43 | 0.66 | 0.22 | 0.12 | 0.22 |
|  | C | 0.31 | 0.26 | 2.86 | 0.81 | 0.30 | 0.20 | 0.14 |
|  | D | 0.36 | 0.30 | 1.75 | 0.58 | 0.25 | 0.34 |  |
|  | E | 0.26 | 0.23 | 1.19 | 0.25 | 0.27 | 0.19 | 0.04 |
|  | F | 0.91 | 0.50 | 0.62 | 0.09 | 0.07 | 0.15 |  |
|  | G | 0.20 | 0.18 | 0.82 | 0.39 | 0.02 | 0.30 | 0.12 |
|  | H | 0.27 | 0.11 | 0.70 | 0.15 | 0.15 | 0.20 |  |
|  | I | 0.40 | 0.20 | 1.14 | 0.49 | 0.13 | 0.19 | 0.15 |
| HSP1A1 | A | 0.18 | 0.26 | 0.55 | 0.27 | 0.12 | 0.18 | 0.21 |
|  | B | 0.16 | 0.65 | 0.23 | 0.32 | 0.67 | 0.18 | 0.17 |
|  | C | 0.72 | 0.13 | 0.66 | 0.22 | 0.42 | 0.24 | 0.25 |
|  | D | 0.32 | 1.58 | 1.42 | 2.58 | 2.26 | 0.83 |  |
|  | E | 1.34 | 1.22 | 1.50 | 8.32 | 1.13 | 0.50 | 0.46 |
|  | F | 0.36 | 0.32 | 1.60 | 2.86 | 0.76 | 0.20 |  |
|  | G | 0.89 | 2.20 | 1.43 | 0.70 | 2.76 | 5.44 | 2.75 |
|  | H | 1.28 | 2.58 | 0.66 | 4.60 | 8.19 | 2.47 |  |
|  | I | 0.51 | 3.27 | 0.85 | 7.56 | 2.16 | 0.87 | 1.05 |
| SDHB | A | 0.45 | 0.46 | 0.57 | 0.40 | 0.26 | 0.35 | 0.43 |
|  | B | 0.41 | 0.82 | 0.30 | 0.65 | 0.63 | 0.24 | 0.34 |
|  | C | 0.66 | 0.30 | 0.44 | 0.59 | 0.82 | 0.52 | 0.35 |
|  | D | 0.37 | 0.89 | 0.28 | 1.14 | 1.53 | 0.76 |  |
|  | E | 0.63 | 0.68 | 1.06 | 1.49 | 0.70 | 0.34 | 0.22 |
|  | F | 0.56 | 0.52 | 0.77 | 1.25 | 0.59 | 0.28 |  |
|  | G | 0.74 | 1.14 | 0.42 | 0.65 | 1.07 | 2.86 | 1.76 |
|  | H | 0.85 | 0.91 | 0.72 | 0.82 | 1.68 | 1.29 |  |
|  | I | 0.86 | 1.48 | 0.31 | 3.50 | 0.87 | 0.64 | 0.26 |
| COX4-1 | A | 3.34 | 4.67 | 8.74 | 5.38 | 3.79 | 2.80 | 3.96 |
|  | B | 3.13 | 11.73 | 3.81 | 6.21 | 9.70 | 2.77 | 3.66 |
|  | C | 7.21 | 3.17 | 5.63 | 5.50 | 9.65 | 5.51 | 4.71 |
|  | D | 4.91 | 22.18 | 7.02 | 17.82 | 26.12 | 12.28 |  |
|  | E | 8.66 | 12.30 | 22.69 | 26.49 | 16.98 | 5.44 | 3.67 |
|  | F | 6.81 | 6.22 | 17.04 | 13.14 | 6.52 | 3.52 |  |
|  | G | 9.63 | 19.11 | 9.87 | 7.01 | 17.53 | 57.21 | 31.53 |
|  | H | 10.08 | 20.16 | 9.33 | 13.06 | 43.01 | 21.03 |  |
|  | I | 8.85 | 40.02 | 7.88 | 70.12 | 9.18 | 7.72 | 3.45 |
| TFAM | A | 0.14 | 0.15 | 0.19 | 0.14 | 0.11 | 0.16 | 0.17 |
|  | B | 0.14 | 0.20 | 0.15 | 0.21 | 0.24 | 0.11 | 0.08 |
|  | C | 0.17 | 0.12 | 0.22 | 0.20 | 0.15 | 0.14 | 0.10 |
|  | D | 0.19 | 0.16 | 0.21 | 0.31 | 0.18 | 0.31 | 1.00 |
|  | E | 0.19 | 0.19 | 0.16 | 0.28 | 0.30 | 0.17 | 0.07 |
|  | F | 0.23 | 0.26 | 0.26 | 0.25 | 0.44 | 0.25 |  |
|  | G | 0.19 | 0.17 | 0.26 | 0.18 | 0.31 | 0.20 | 0.17 |
|  | H | 0.24 | 0.19 | 0.25 | 0.37 | 0.28 | 0.22 |  |
|  | I | 0.24 | 0.17 | 0.21 | 0.30 | 0.32 | 0.27 | 0.09 |
| CS | A | 0.33 | 0.43 | 1.31 | 0.65 | 0.51 | 0.50 | 0.56 |
|  | B | 0.58 | 1.44 | 0.49 | 0.56 | 0.82 | 0.43 | 0.53 |
|  | C | 1.02 | 0.39 | 0.77 | 0.72 | 1.32 | 0.56 | 0.15 |
|  | D | 0.59 | 1.17 | 0.53 | 1.07 | 1.90 | 1.15 |  |
|  | E | 0.69 | 0.66 | 1.34 | 1.68 | 1.12 | 0.33 | 0.26 |
|  | F | 0.87 | 0.52 | 1.02 | 1.36 | 0.72 | 0.67 |  |
|  | G | 0.64 | 1.39 | 0.68 | 0.65 | 1.35 | 4.83 | 3.05 |
|  | H | 0.72 | 1.09 | 0.71 | 0.67 | 2.94 | 1.57 |  |
|  | I | 0.66 | 2.63 | 0.50 | 4.77 | 0.58 | 0.49 | 0.21 |
| p53 | A | 0.05 | 0.04 | 0.18 | 0.10 | 0.08 | 0.15 | 0.06 |
|  | B | 0.08 | 0.07 | 0.09 | 0.07 | 0.18 | 0.20 | 0.14 |
|  | C | 0.08 | 0.08 | 0.09 | 0.11 | 0.11 | 0.27 | 0.18 |
|  | D | 0.05 | 0.05 | 0.04 | 0.07 | 0.09 | 0.07 |  |
|  | E | 0.05 | 0.03 | 0.05 | 0.09 | 0.14 | 0.21 | 0.21 |
|  | F | 0.07 | 0.08 | 0.08 | 0.15 | 0.20 | 0.46 |  |
|  | G | 0.08 | 0.09 | 0.07 | 0.06 | 0.17 | 0.09 | 0.18 |
|  | H | 0.05 | 0.07 | 0.08 | 0.23 | 0.12 | 0.05 |  |
|  | I | 0.05 | 0.07 | 0.09 | 0.07 | 0.29 | 0.08 | 0.09 |
| GLUT4 | A | 0.72 | 0.30 | 0.96 | 0.39 | 0.47 | 0.24 | 0.42 |
|  | B | 0.83 | 0.60 | 0.54 | 0.74 | 0.46 | 0.21 | 0.38 |
|  | C | 0.93 | 0.33 | 0.56 | 0.85 | 0.62 | 0.41 | 0.40 |
|  | D | 0.48 | 0.50 | 0.62 | 0.79 | 0.63 | 0.70 |  |
|  | E | 0.72 | 0.37 | 0.51 | 0.47 | 0.36 | 0.06 | 0.02 |
|  | F | 1.54 | 0.71 | 0.24 | 0.45 | 0.21 | 0.10 |  |
|  | G | 0.52 | 0.37 | 0.36 | 0.60 | 0.02 | 0.59 | 0.32 |
|  | H | 0.76 | 0.26 | 0.57 | 0.49 | 0.53 | 0.50 |  |
|  | I | 1.08 | 0.48 | 0.44 | 0.83 | 0.23 | 0.30 | 0.24 |
| CPT1A | A | 0.27 | 0.19 | 0.84 | 0.45 | 0.12 | 0.08 | 0.15 |
|  | B | 0.17 | 0.19 | 1.07 | 0.47 | 0.12 | 0.08 | 0.14 |
|  | C | 0.04 | 0.02 | 0.02 | 0.05 | 0.02 | 0.02 | 0.04 |
|  | D | 0.16 | 0.14 | 0.81 | 0.38 | 0.16 | 0.19 |  |
|  | E | 0.10 | 0.12 | 0.76 | 0.15 | 0.19 | 0.07 | 0.03 |
|  | F | 0.05 | 0.06 | 0.03 | 0.04 | 0.04 | 0.08 |  |
|  | G | 0.18 | 0.11 | 1.34 | 0.24 | 0.02 | 0.20 | 0.15 |
|  | H | 0.22 | 0.09 | 0.46 | 0.19 | 0.13 | 0.16 |  |
|  | I | 0.34 | 0.15 | 1.21 | 0.48 | 0.09 | 0.18 | 0.10 |
| NDUFB3 | A | 0.69 | 0.79 | 1.21 | 0.66 | 0.47 | 0.54 | 0.63 |
|  | B | 0.63 | 1.24 | 0.69 | 1.10 | 1.02 | 0.47 | 0.46 |
|  | C | 1.13 | 0.33 | 1.25 | 0.60 | 1.33 | 0.71 | 0.54 |
|  | D | 0.77 | 3.11 | 1.31 | 4.51 | 6.35 | 2.54 |  |
|  | E | 1.94 | 2.66 | 5.36 | 7.32 | 3.02 | 1.04 | 0.46 |
|  | F | 0.86 | 0.80 | 3.87 | 3.49 | 1.11 | 0.50 |  |
|  | G | 1.76 | 4.24 | 1.71 | 1.38 | 3.95 | 12.65 | 4.20 |
|  | H | 2.59 | 5.02 | 2.74 | 4.46 | 14.16 | 3.32 |  |
|  | I | 2.20 | 10.25 | 1.44 | 13.98 | 1.66 | 2.03 | 0.92 |
| PDK4 | A | 0.43 | 0.45 | 1.44 | 0.34 | 0.43 | 0.41 | 0.13 |
|  | B | 0.40 | 0.02 | 0.21 | 0.06 | 0.08 | 0.19 | 0.02 |
|  | C | 0.61 | 0.01 | 0.43 | 0.19 | 0.14 | 0.25 | 0.32 |
|  | D | 0.04 | 0.31 | 0.35 | 0.66 | 1.36 | 0.20 |  |
|  | E | 0.11 | 0.21 | 1.30 | 7.69 | 1.06 | 0.78 | 0.29 |
|  | F | 0.16 | 0.06 | 0.89 | 13.37 | 2.73 | 0.57 |  |
|  | G | 0.13 | 0.19 | 0.42 | 0.38 | 3.02 | 0.47 | 3.21 |
|  | H | 0.04 | 0.38 | 0.19 | 7.71 | 2.73 | 0.12 |  |
|  | I | 0.14 | 1.47 | 0.52 | 5.05 | 2.00 | 0.28 | 0.20 |
| VEGF | A | 0.10 | 0.11 | 0.33 | 0.19 | 0.11 | 0.04 | 0.11 |
|  | B | 0.13 | 0.21 | 0.14 | 0.22 | 0.11 | 0.03 | 0.09 |
|  | C | 0.16 | 0.08 | 0.23 | 0.21 | 0.07 | 0.04 | 0.03 |
|  | D | 0.19 | 0.10 | 0.26 | 0.29 | 0.13 | 0.18 |  |
|  | E | 0.11 | 0.11 | 0.13 | 0.14 | 0.06 | 0.02 | 0.02 |
|  | F | 0.65 | 0.26 | 0.12 | 0.22 | 0.10 | 0.03 |  |
|  | G | 0.11 | 0.07 | 0.14 | 0.14 | 0.03 | 0.11 | 0.05 |
|  | H | 0.15 | 0.05 | 0.16 | 0.15 | 0.10 | 0.10 |  |
|  | I | 0.14 | 0.09 | 0.18 | 0.28 | 0.09 | 0.09 | 0.04 |
| PGC-1β | A | 0.37 | 0.19 | 0.97 | 0.91 | 0.44 | 0.11 | 0.37 |
|  | B | 0.56 | 0.82 | 0.63 | 1.14 | 0.46 | 0.41 | 0.85 |
|  | C | 0.79 | 0.58 | 0.60 | 1.30 | 0.62 | 0.73 | 0.51 |
|  | D | 0.64 | 0.40 | 0.57 | 1.24 | 0.60 | 0.89 |  |
|  | E | 0.35 | 0.32 | 0.38 | 0.17 | 0.34 | 0.13 | 0.23 |
|  | F | 0.53 | 0.56 | 0.30 | 0.16 | 0.12 | 0.51 |  |
|  | G | 0.54 | 0.48 | 0.46 | 0.79 | 0.09 | 0.62 | 0.36 |
|  | H | 1.09 | 0.45 | 0.79 | 0.17 | 0.51 | 0.53 |  |
|  | I | 0.55 | 0.39 | 0.68 | 1.09 | 0.17 | 0.31 | 0.32 |
| NRF1 | A | 0.04 | 0.07 | 0.18 | 0.16 | 0.10 | 0.19 | 0.08 |
|  | B | 0.08 | 0.07 | 0.09 | 0.07 | 0.18 | 0.20 | 0.14 |
|  | C | 0.08 | 0.08 | 0.09 | 0.11 | 0.11 | 0.27 | 0.18 |
|  | D | 0.05 | 0.05 | 0.04 | 0.07 | 0.09 | 0.07 |  |
|  | E | 0.05 | 0.03 | 0.05 | 0.09 | 0.14 | 0.21 | 0.21 |
|  | F | 0.07 | 0.08 | 0.08 | 0.15 | 0.20 | 0.46 |  |
|  | G | 0.08 | 0.09 | 0.07 | 0.06 | 0.17 | 0.09 | 0.18 |
|  | H | 0.05 | 0.07 | 0.08 | 0.23 | 0.12 | 0.05 |  |
|  | I | 0.05 | 0.07 | 0.09 | 0.07 | 0.29 | 0.08 | 0.09 |
| CD36 | A | 19.26 | 18.42 | 13.99 | 8.41 | 8.63 | 1.07 | 1.70 |
|  | B | 1.71 | 1.50 | 1.51 | 1.32 | 0.30 | 0.17 | 1.55 |
|  | C | 1.74 | 1.39 | 1.04 | 1.08 | 0.36 | 0.19 | 0.77 |
|  | D | 4.35 | 9.73 | 13.39 | 3.19 | 1.43 | 3.57 | 1.00 |
|  | E | 3.94 | 2.00 | 3.38 | 1.49 | 3.04 | 0.33 | 0.23 |
|  | F | 3.95 | 2.72 | 3.40 | 0.60 | 0.58 | 0.21 |  |
|  | G | 3.86 | 5.18 | 4.45 | 5.20 | 0.41 | 1.16 | 1.10 |
|  | H | 2.89 | 2.47 | 5.73 | 2.83 | 1.69 | 0.62 |  |
|  | I | 19.02 | 10.63 | 13.97 | 10.78 | 2.50 | 2.27 | 5.05 |
| TFEB | A | 0.13 | 0.13 | 0.39 | 0.17 | 0.10 | 0.15 | 0.13 |
|  | B | 0.20 | 0.09 | 0.05 | 0.07 | 0.10 | 0.11 | 0.13 |
|  | C | 0.16 | 0.09 | 0.11 | 0.17 | 0.09 | 0.16 | 0.19 |
|  | D | 0.21 | 0.10 | 0.16 | 0.17 | 0.16 | 0.20 | 1.00 |
|  | E | 0.11 | 0.07 | 0.11 | 0.09 | 0.11 | 0.12 | 0.14 |
|  | F | 0.12 | 0.11 | 0.04 | 0.08 | 0.05 | 0.22 |  |
|  | G | 0.12 | 0.07 | 0.12 | 0.11 | 0.04 | 0.12 | 0.11 |
|  | H | 0.15 | 0.12 | 0.16 | 0.20 | 0.17 | 0.20 |  |
|  | I | 0.15 | 0.10 | 0.07 | 0.14 | 0.06 | 0.15 | 0.08 |
| UCP3 | A | 0.09 | 0.11 | 0.15 | 0.07 | 0.10 | 0.12 | 0.11 |
|  | B | 0.06 | 0.02 | 0.01 | 0.04 | 0.03 | 0.10 | 0.01 |
|  | C | 0.17 | 0.01 | 0.03 | 0.05 | 0.12 | 0.08 | 0.11 |
|  | D | 0.02 | 0.09 | 0.06 | 0.11 | 0.47 | 0.14 |  |
|  | E | 0.03 | 0.04 | 0.13 | 0.08 | 0.24 | 0.05 | 0.05 |
|  | F | 0.05 | 0.03 | 0.10 | 0.09 | 0.10 | 0.11 |  |
|  | G | 0.08 | 0.17 | 0.14 | 0.07 | 0.14 | 0.35 | 0.54 |
|  | H | 0.02 | 0.18 | 0.09 | 0.06 | 0.28 | 0.05 |  |
|  | I | 0.13 | 0.33 | 0.05 | 0.19 | 0.07 | 0.15 | 0.08 |
| UQCRC2 | A | 0.22 | 0.22 | 0.43 | 0.19 | 0.12 | 0.16 | 0.14 |
|  | B | 0.22 | 0.40 | 0.14 | 0.18 | 0.20 | 0.09 | 0.10 |
|  | C | 0.36 | 0.13 | 0.25 | 0.19 | 0.43 | 0.18 | 0.17 |
|  | D | 0.21 | 0.47 | 0.20 | 0.41 | 0.76 | 0.36 |  |
|  | E | 0.24 | 0.33 | 0.51 | 0.52 | 0.34 | 0.13 | 0.07 |
|  | F | 0.28 | 0.23 | 0.37 | 0.43 | 0.30 | 0.14 |  |
|  | G | 0.18 | 0.51 | 0.21 | 0.18 | 0.51 | 0.69 | 0.78 |
|  | H | 0.28 | 0.38 | 0.30 | 0.29 | 0.82 | 0.39 |  |
|  | I | 0.42 | 1.02 | 0.17 | 0.84 | 0.35 | 0.19 | 0.11 |
| PPARβ/δ | A | 0.37 | 0.24 | 0.77 | 0.28 | 0.26 | 0.81 |  |
|  | B | 0.56 | 0.18 | 0.24 | 0.23 | 0.22 | 0.17 | 0.20 |
|  | C | 0.19 | 0.07 | 0.09 | 0.16 | 0.20 | 0.24 | 0.16 |
|  | D | 0.24 | 0.17 | 0.24 | 0.22 | 0.32 | 0.31 |  |
|  | E | 0.36 | 0.17 | 0.24 | 0.41 | 0.39 | 0.22 | 0.48 |
|  | F | 0.20 | 0.15 | 0.07 | 0.56 | 0.31 | 0.36 |  |
|  | G | 0.29 | 0.23 | 0.30 | 0.24 | 0.54 | 0.34 | 0.83 |
|  | H | 0.55 | 0.27 | 0.52 | 1.24 | 0.94 | 0.37 |  |
|  | I | 0.22 | 0.18 | 0.22 | 0.20 | 0.31 | 0.34 | 0.18 |
| PPARγ | A | 0.13 | 0.07 | 0.08 | 0.07 | 0.09 | 0.05 | 0.15 |
|  | B | 0.05 | 0.05 | 0.04 | 0.07 | 0.04 | 0.05 | 0.10 |
|  | C | 0.03 | 0.05 | 0.04 | 0.07 | 0.06 | 0.08 | 0.05 |
|  | D | 0.04 | 0.06 | 0.05 | 0.06 | 0.08 | 0.11 |  |
|  | E | 0.04 | 0.04 | 0.04 | 0.02 | 0.07 | 0.05 | 0.05 |
|  | F | 0.05 | 0.09 | 0.05 | 0.05 | 0.13 | 0.08 |  |
|  | G | 0.01 | 0.03 | 0.03 | 0.03 | 0.06 | 0.05 | 0.08 |
|  | H | 0.03 | 0.05 | 0.06 | 0.04 | 0.06 | 0.06 |  |
|  | I | 0.04 | 0.05 | 0.20 | 0.06 | 0.07 | 0.08 | 0.03 |
| MFN2 | A | 0.83 | 0.65 | 1.32 | 1.48 | 1.21 | 0.79 | 3.37 |
|  | B | 0.84 | 0.85 | 0.82 | 0.97 | 1.06 | 0.98 | 1.52 |
|  | C | 0.76 | 0.38 | 0.77 | 0.87 | 0.92 | 1.22 | 1.61 |
|  | D | 1.38 | 3.77 | 3.32 | 1.11 | 2.26 | 7.37 |  |
|  | E | 1.59 | 1.30 | 1.62 | 1.27 | 6.05 | 0.97 | 1.00 |
|  | F | 1.50 | 1.32 | 0.34 | 1.03 | 1.44 | 1.96 |  |
|  | G | 1.65 | 0.99 | 1.96 | 1.52 | 0.82 | 2.84 | 1.41 |
|  | H | 1.22 | 0.63 | 1.21 | 1.93 | 1.76 | 1.19 |  |
