# Supplementary material for: Interpretation of exercise-induced changes in human skeletal muscle mRNA expression depends on the timing of the post-exercise biopsies
Source: PeerJ. 2022 Feb 4;10:e12856. doi: 10.7717/peerj.12856 (PMC8820226; doi:10.7717/peerj.12856)
Supplement: Supplemental Information 3 [file peerj-10-12856-s003.docx]

S3 Table: Fold change of all target mRNA expression relative to baseline.

| Gene | Participant | Fold change relative to baseline | | | | | |
| --- | --- | --- | --- | --- | --- | --- | --- |
|  |  | 0 h | 3 h | 9 h | 24 h | 48 h | 72 h |
| PGC-1α | A | 0.78 | 2.76 | 1.24 | 0.47 | 0.26 | 0.41 |
|  | B | 1.25 | 2.78 | 1.61 | 0.54 | 0.28 | 0.86 |
|  | C | 0.76 | 6.89 | 2.71 | 1.52 | 0.73 | 0.46 |
|  | D | 1.25 | 4.66 | 2.31 | 1.26 | 1.35 |  |
|  | E | 0.97 | 4.12 | 0.85 | 1.35 | 0.85 | 0.21 |
|  | F | 0.81 | 0.9 | 0.18 | 0.18 | 0.33 |  |
|  | G | 1.01 | 4.99 | 1.43 | 0.11 | 1.45 | 0.77 |
|  | H | 0.52 | 2.94 | 0.81 | 0.71 | 0.94 |  |
|  | I | 0.41 | 2.53 | 1.01 | 0.45 | 0.47 | 0.22 |
| PGC-1α4 | A | 0.70 | 3.15 | 1.70 | 0.44 | 0.28 | 0.56 |
|  | B | 1.13 | 6.30 | 2.76 | 0.68 | 0.50 | 0.81 |
|  | C | 0.87 | 17.87 | 2.87 | 1.36 | 0.91 | 0.56 |
|  | D | 0.89 | 5.17 | 2.43 | 1.01 | 1.21 |  |
|  | E | 1.13 | 7.32 | 1.43 | 1.79 | 0.68 | 0.31 |
|  | F | 1.25 | 0.96 | 0.31 | 0.30 | 0.49 |  |
|  | G | 0.60 | 7.32 | 1.33 | 0.13 | 1.09 | 0.81 |
|  | H | 0.40 | 2.13 | 0.86 | 0.61 | 0.72 |  |
|  | I | 0.44 | 3.54 | 1.40 | 0.26 | 0.52 | 0.29 |
| PPARα | A | 0.96 | 4.54 | 1.06 | 0.77 | 0.45 | 0.82 |
|  | B | 1.2 | 4.36 | 2.02 | 0.68 | 0.37 | 0.66 |
|  | C | 0.84 | 9.28 | 2.63 | 0.96 | 0.64 | 0.46 |
|  | D | 0.85 | 4.92 | 1.62 | 0.69 | 0.95 |  |
|  | E | 0.88 | 4.64 | 0.97 | 1.03 | 0.74 | 0.16 |
|  | F | 1.42 | 2.61 | 0.22 | 0.24 | 0.46 |  |
|  | G | 0.91 | 4.04 | 1.89 | 0.09 | 1.49 | 0.61 |
|  | H | 0.39 | 2.6 | 0.57 | 0.54 | 0.75 |  |
|  | I | 0.49 | 2.84 | 1.23 | 0.33 | 0.48 | 0.38 |
| HSP1A1 | A | 1.47 | 3.04 | 1.52 | 0.69 | 1 | 1.14 |
|  | B | 4.1 | 1.47 | 2.03 | 4.22 | 1.12 | 1.09 |
|  | C | 0.19 | 0.92 | 0.31 | 0.59 | 0.33 | 0.35 |
|  | D | 4.96 | 4.45 | 8.08 | 7.08 | 2.58 |  |
|  | E | 0.91 | 1.12 | 6.2 | 0.84 | 0.37 | 0.34 |
|  | F | 0.87 | 4.42 | 7.88 | 2.11 | 0.56 |  |
|  | G | 2.47 | 1.61 | 0.79 | 3.1 | 6.12 | 3.09 |
|  | H | 2.02 | 0.51 | 3.61 | 6.41 | 1.93 |  |
|  | I | 6.39 | 1.66 | 14.76 | 4.21 | 1.69 | 2.05 |
| SDHB | A | 1.02 | 1.26 | 0.88 | 0.57 | 0.77 | 0.95 |
|  | B | 1.99 | 0.74 | 1.58 | 1.53 | 0.59 | 0.83 |
|  | C | 0.45 | 0.67 | 0.89 | 1.25 | 0.79 | 0.53 |
|  | D | 2.43 | 0.78 | 3.1 | 4.17 | 2.07 |  |
|  | E | 1.08 | 1.69 | 2.37 | 1.12 | 0.54 | 0.35 |
|  | F | 0.93 | 1.36 | 2.21 | 1.05 | 0.5 |  |
|  | G | 1.54 | 0.56 | 0.88 | 1.45 | 3.85 | 2.37 |
|  | H | 1.07 | 0.84 | 0.97 | 1.98 | 1.52 |  |
|  | I | 1.72 | 0.36 | 4.05 | 1.01 | 0.75 | 0.3 |
| COX4-1 | A | 1.4 | 2.61 | 1.61 | 1.13 | 0.84 | 1.18 |
|  | B | 3.75 | 1.22 | 1.99 | 3.1 | 0.89 | 1.17 |
|  | C | 0.44 | 0.78 | 0.76 | 1.34 | 0.76 | 0.65 |
|  | D | 4.52 | 1.43 | 3.63 | 5.32 | 2.5 |  |
|  | E | 1.42 | 2.62 | 3.06 | 1.96 | 0.63 | 0.42 |
|  | F | 0.91 | 2.5 | 1.93 | 0.96 | 0.52 |  |
|  | G | 1.98 | 1.03 | 0.73 | 1.82 | 5.94 | 3.27 |
|  | H | 2 | 0.93 | 1.3 | 4.27 | 2.09 |  |
|  | I | 4.52 | 0.89 | 7.92 | 1.04 | 0.87 | 0.39 |
| TFAM | A | 1.02 | 1.3 | 0.95 | 0.76 | 1.09 | 1.2 |
|  | B | 1.41 | 1.07 | 1.46 | 1.66 | 0.77 | 0.54 |
|  | C | 0.69 | 1.3 | 1.2 | 0.92 | 0.8 | 0.56 |
|  | D | 0.84 | 1.12 | 1.65 | 0.93 | 1.62 |  |
|  | E | 1.02 | 0.87 | 1.53 | 1.61 | 0.94 | 0.36 |
|  | F | 1.12 | 1.12 | 1.07 | 1.85 | 1.05 |  |
|  | G | 0.94 | 1.42 | 0.96 | 1.69 | 1.08 | 0.92 |
|  | H | 0.78 | 1.01 | 1.51 | 1.17 | 0.89 |  |
|  | I | 0.7 | 0.87 | 1.25 | 1.32 | 1.11 | 0.39 |
| CS | A | 1.32 | 4 | 1.99 | 1.55 | 1.53 | 1.72 |
|  | B | 2.48 | 0.83 | 0.97 | 1.4 | 0.74 | 0.91 |
|  | C | 0.38 | 0.76 | 0.71 | 1.29 | 0.55 | 0.14 |
|  | D | 2 | 0.91 | 1.83 | 3.23 | 1.95 |  |
|  | E | 0.96 | 1.95 | 2.44 | 1.63 | 0.48 | 0.38 |
|  | F | 0.6 | 1.17 | 1.56 | 0.82 | 0.77 |  |
|  | G | 2.17 | 1.05 | 1.01 | 2.11 | 7.53 | 4.76 |
|  | H | 1.53 | 1 | 0.94 | 4.1 | 2.19 |  |
|  | I | 3.99 | 0.76 | 7.24 | 0.87 | 0.74 | 0.32 |
| p53 | A | 0.74 | 3.67 | 2.1 | 1.68 | 2.94 | 1.17 |
|  | B | 0.88 | 1.24 | 0.91 | 2.4 | 2.65 | 1.79 |
|  | C | 1 | 1.18 | 1.42 | 1.43 | 3.36 | 2.33 |
|  | D | 0.96 | 0.81 | 1.33 | 1.77 | 1.36 |  |
|  | E | 0.52 | 1.09 | 1.8 | 2.72 | 4.25 | 4.28 |
|  | F | 1.12 | 1.16 | 2.22 | 2.95 | 6.7 |  |
|  | G | 1.04 | 0.9 | 0.69 | 2.13 | 1.14 | 2.18 |
|  | H | 1.25 | 1.41 | 4.13 | 2.18 | 0.95 |  |
|  | I | 1.43 | 1.67 | 1.32 | 5.61 | 1.53 | 1.72 |
| GLUT4 | A | 0.42 | 1.33 | 0.54 | 0.65 | 0.33 | 0.59 |
|  | B | 0.72 | 0.64 | 0.88 | 0.55 | 0.25 | 0.46 |
|  | C | 0.35 | 0.59 | 0.9 | 0.67 | 0.44 | 0.43 |
|  | D | 1.03 | 1.29 | 1.64 | 1.32 | 1.46 |  |
|  | E | 0.52 | 0.72 | 0.65 | 0.5 | 0.09 | 0.02 |
|  | F | 0.46 | 0.16 | 0.29 | 0.14 | 0.06 |  |
|  | G | 0.71 | 0.68 | 1.15 | 0.05 | 1.13 | 0.61 |
|  | H | 0.34 | 0.75 | 0.65 | 0.69 | 0.65 |  |
|  | I | 0.44 | 0.41 | 0.77 | 0.21 | 0.28 | 0.22 |
| CPT1A | A | 0.7 | 3.15 | 1.7 | 0.44 | 0.28 | 0.56 |
|  | B | 1.13 | 6.3 | 2.76 | 0.68 | 0.5 | 0.81 |
|  | C | 0.42 | 0.49 | 1.1 | 0.38 | 0.58 | 0.98 |
|  | D | 0.89 | 5.17 | 2.43 | 1.01 | 1.21 |  |
|  | E | 1.13 | 7.32 | 1.43 | 1.79 | 0.68 | 0.31 |
|  | F | 1.15 | 0.63 | 0.7 | 0.76 | 1.57 |  |
|  | G | 0.6 | 7.32 | 1.33 | 0.13 | 1.09 | 0.81 |
|  | H | 0.4 | 2.13 | 0.86 | 0.61 | 0.72 |  |
|  | I | 0.44 | 3.54 | 1.4 | 0.26 | 0.52 | 0.29 |
| NDUFB3 | A | 1.14 | 1.74 | 0.95 | 0.68 | 0.77 | 0.91 |
|  | B | 1.98 | 1.11 | 1.77 | 1.63 | 0.76 | 0.74 |
|  | C | 0.29 | 1.11 | 0.54 | 1.18 | 0.63 | 0.48 |
|  | D | 4.07 | 1.72 | 5.9 | 8.3 | 3.32 |  |
|  | E | 1.38 | 2.77 | 3.78 | 1.56 | 0.54 | 0.24 |
|  | F | 0.92 | 4.49 | 4.05 | 1.29 | 0.58 |  |
|  | G | 2.41 | 0.97 | 0.78 | 2.24 | 7.19 | 2.39 |
|  | H | 1.94 | 1.06 | 1.73 | 5.48 | 1.28 |  |
|  | I | 4.66 | 0.65 | 6.36 | 0.76 | 0.92 | 0.42 |
| PDK4 | A | 1.05 | 3.35 | 0.79 | 1.01 | 0.94 | 0.31 |
|  | B | 0.05 | 0.53 | 0.15 | 0.21 | 0.47 | 0.05 |
|  | C | 0.02 | 0.7 | 0.31 | 0.23 | 0.41 | 0.52 |
|  | D | 6.99 | 7.89 | 14.99 | 30.72 | 4.57 |  |
|  | E | 1.93 | 11.68 | 69.26 | 9.54 | 6.99 | 2.59 |
|  | F | 0.37 | 5.66 | 85.48 | 17.45 | 3.66 |  |
|  | G | 1.51 | 3.26 | 2.94 | 23.49 | 3.66 | 24.97 |
|  | H | 9.63 | 4.94 | 195.83 | 69.28 | 2.94 |  |
|  | I | 10.19 | 3.62 | 34.91 | 13.85 | 1.96 | 1.36 |
| VEGF | A | 1.15 | 3.37 | 1.99 | 1.16 | 0.44 | 1.17 |
|  | B | 1.63 | 1.12 | 1.73 | 0.83 | 0.23 | 0.74 |
|  | C | 0.54 | 1.48 | 1.33 | 0.42 | 0.28 | 0.18 |
|  | D | 0.56 | 1.4 | 1.57 | 0.7 | 0.97 |  |
|  | E | 1.04 | 1.19 | 1.27 | 0.53 | 0.15 | 0.15 |
|  | F | 0.39 | 0.19 | 0.34 | 0.16 | 0.05 |  |
|  | G | 0.67 | 1.35 | 1.3 | 0.31 | 1.05 | 0.45 |
|  | H | 0.35 | 1.11 | 1.01 | 0.68 | 0.67 |  |
|  | I | 0.65 | 1.23 | 1.95 | 0.65 | 0.62 | 0.25 |
| PGC-1β | A | 0.52 | 2.63 | 2.48 | 1.19 | 0.3 | 1.01 |
|  | B | 1.47 | 1.12 | 2.05 | 0.82 | 0.73 | 1.53 |
|  | C | 0.73 | 0.76 | 1.65 | 0.78 | 0.93 | 0.65 |
|  | D | 0.63 | 0.88 | 1.92 | 0.94 | 1.38 |  |
|  | E | 0.89 | 1.06 | 0.48 | 0.96 | 0.36 | 0.66 |
|  | F | 1.06 | 0.57 | 0.3 | 0.23 | 0.97 |  |
|  | G | 0.88 | 0.86 | 1.45 | 0.17 | 1.15 | 0.67 |
|  | H | 0.42 | 0.72 | 0.15 | 0.47 | 0.48 |  |
|  | I | 0.71 | 1.24 | 1.98 | 0.31 | 0.56 | 0.58 |
| NRF1 | A | 2.07 | 5.11 | 4.31 | 2.83 | 5.2 | 2.25 |
|  | B | 0.88 | 1.24 | 0.91 | 2.4 | 2.65 | 1.79 |
|  | C | 1 | 1.18 | 1.42 | 1.43 | 3.36 | 2.33 |
|  | D | 0.96 | 0.81 | 1.33 | 1.77 | 1.36 |  |
|  | E | 0.52 | 1.09 | 1.8 | 2.72 | 4.25 | 4.28 |
|  | F | 1.12 | 1.16 | 2.22 | 2.95 | 6.7 |  |
|  | G | 1.04 | 0.9 | 0.69 | 2.13 | 1.14 | 2.18 |
|  | H | 1.25 | 1.41 | 4.13 | 2.18 | 0.95 |  |
|  | I | 1.43 | 1.67 | 1.32 | 5.61 | 1.53 | 1.72 |
| CD36 | A | 0.96 | 0.73 | 0.44 | 0.45 | 0.06 | 0.09 |
|  | B | 0.88 | 0.88 | 0.77 | 0.18 | 0.1 | 0.91 |
|  | C | 0.8 | 0.6 | 0.62 | 0.21 | 0.11 | 0.44 |
|  | D | 2.24 | 3.08 | 0.73 | 0.33 | 0.82 |  |
|  | E | 0.51 | 0.86 | 0.38 | 0.77 | 0.08 | 0.06 |
|  | F | 0.69 | 0.86 | 0.15 | 0.15 | 0.05 |  |
|  | G | 1.34 | 1.15 | 1.35 | 0.11 | 0.3 | 0.28 |
|  | H | 0.85 | 1.98 | 0.98 | 0.59 | 0.22 |  |
|  | I | 0.56 | 0.73 | 0.57 | 0.13 | 0.12 | 0.27 |
| TFEB | A | 1.01 | 2.95 | 1.31 | 0.74 | 1.17 | 0.97 |
|  | B | 0.46 | 0.27 | 0.35 | 0.51 | 0.55 | 0.66 |
|  | C | 0.56 | 0.69 | 1.05 | 0.57 | 0.96 | 1.17 |
|  | D | 0.49 | 0.74 | 0.82 | 0.75 | 0.98 |  |
|  | E | 0.61 | 1 | 0.79 | 1.04 | 1.07 | 1.31 |
|  | F | 0.97 | 0.34 | 0.66 | 0.42 | 1.86 |  |
|  | G | 0.59 | 0.99 | 0.89 | 0.35 | 0.97 | 0.95 |
|  | H | 0.81 | 1.08 | 1.34 | 1.15 | 1.35 |  |
|  | I | 0.67 | 0.48 | 0.89 | 0.4 | 0.99 | 0.53 |
| UCP3 | A | 1.28 | 1.72 | 0.76 | 1.08 | 1.36 | 1.23 |
|  | B | 0.28 | 0.22 | 0.73 | 0.53 | 1.71 | 0.23 |
|  | C | 0.06 | 0.19 | 0.28 | 0.7 | 0.5 | 0.67 |
|  | D | 4.99 | 3.35 | 6.35 | 25.92 | 7.91 |  |
|  | E | 1.46 | 4.76 | 3.17 | 9.26 | 1.94 | 1.99 |
|  | F | 0.61 | 1.86 | 1.77 | 1.89 | 2.2 |  |
|  | G | 1.98 | 1.68 | 0.78 | 1.69 | 4.16 | 6.43 |
|  | H | 9.43 | 4.49 | 3.24 | 14.2 | 2.79 |  |
|  | I | 2.57 | 0.42 | 1.49 | 0.56 | 1.18 | 0.6 |
| UQCRC2 | A | 0.99 | 1.96 | 0.86 | 0.55 | 0.74 | 0.65 |
|  | B | 1.79 | 0.64 | 0.82 | 0.89 | 0.41 | 0.43 |
|  | C | 0.35 | 0.69 | 0.53 | 1.19 | 0.51 | 0.47 |
|  | D | 2.22 | 0.92 | 1.96 | 3.59 | 1.69 |  |
|  | E | 1.37 | 2.13 | 2.18 | 1.44 | 0.56 | 0.29 |
|  | F | 0.82 | 1.3 | 1.53 | 1.07 | 0.5 |  |
|  | G | 2.81 | 1.13 | 0.99 | 2.8 | 3.76 | 4.27 |
|  | H | 1.38 | 1.08 | 1.04 | 2.96 | 1.41 |  |
|  | I | 2.43 | 0.41 | 2 | 0.82 | 0.45 | 0.26 |
| PPARβ/δ | A | 0.65 | 2.08 | 0.77 | 0.71 | 2.17 |  |
|  | B | 0.32 | 0.44 | 0.41 | 0.4 | 0.31 | 0.36 |
|  | C | 0.38 | 0.5 | 0.85 | 1.04 | 1.24 | 0.86 |
|  | D | 0.7 | 1.01 | 0.9 | 1.31 | 1.27 |  |
|  | E | 0.49 | 0.66 | 1.16 | 1.1 | 0.63 | 1.35 |
|  | F | 0.76 | 0.35 | 2.8 | 1.53 | 1.8 |  |
|  | G | 0.8 | 1.05 | 0.84 | 1.88 | 1.17 | 2.9 |
|  | H | 0.49 | 0.95 | 2.25 | 1.7 | 0.68 |  |
|  | I | 0.82 | 1 | 0.94 | 1.44 | 1.57 | 0.8 |
| PPARγ | A | 0.56 | 0.64 | 0.52 | 0.71 | 0.38 | 1.12 |
|  | B | 1.14 | 0.93 | 1.49 | 0.95 | 1.12 | 2.28 |
|  | C | 1.34 | 1.14 | 1.92 | 1.74 | 2.42 | 1.47 |
|  | D | 1.54 | 1.22 | 1.45 | 1.88 | 2.55 |  |
|  | E | 0.92 | 0.97 | 0.43 | 1.59 | 1.21 | 1.11 |
|  | F | 2.04 | 1.14 | 1 | 2.75 | 1.83 |  |
|  | G | 4.02 | 4.32 | 4.24 | 9.3 | 7.53 | 10.96 |
|  | H | 1.62 | 1.95 | 1.52 | 1.91 | 2.1 |  |
|  | I | 1.18 | 4.68 | 1.34 | 1.6 | 1.97 | 0.74 |
| MFN2 | A | 0.78 | 1.59 | 1.78 | 1.45 | 0.95 | 4.06 |
|  | B | 1.01 | 0.98 | 1.16 | 1.27 | 1.17 | 1.82 |
|  | C | 0.51 | 1.01 | 1.15 | 1.22 | 1.61 | 2.12 |
|  | D | 2.73 | 2.41 | 0.8 | 1.64 | 5.35 |  |
|  | E | 0.82 | 1.02 | 0.8 | 3.81 | 0.61 | 0.63 |
|  | F | 0.88 | 0.23 | 0.69 | 0.96 | 1.31 |  |
|  | G | 0.6 | 1.19 | 0.92 | 0.5 | 1.73 | 0.86 |
|  | H | 0.51 | 1 | 1.58 | 1.45 | 0.98 |  |
