# Supplementary material for: Interpretation of exercise-induced changes in human skeletal muscle mRNA expression depends on the timing of the post-exercise biopsies
Source: PeerJ. 2022 Feb 4;10:e12856. doi: 10.7717/peerj.12856 (PMC8820226; doi:10.7717/peerj.12856)
Supplement: Supplemental Information 4 [file peerj-10-12856-s004.docx]

S4 Table: Calculated mRNA expression peak for 8 gene isoforms.

| Gene | Calculated mRNA expression peak (h) | | |
| --- | --- | --- | --- |
|  | Mean | Upper limit | Lower Limit |
| PPARα | 4.59 | 5.69 | 3.62 |
| PGC-1α | 4.7 | 5.94 | 3.49 |
| PGC-1α4 | 4.84 | 6.06 | 3.86 |
| CPT1A | 5.05 | 3.83 | 6.34 |
| PDK4 | 15.27 | 11.95 | 17.97 |
| UCP3 | 31.70 | 21.03 | 43.95 |
| NRF1 | 33.89 | 23.55 | 47.2 |
| p53 | 34.78 | 22.44 | 46.88 |
