# Supplementary material for: Interpretation of exercise-induced changes in human skeletal muscle mRNA expression depends on the timing of the post-exercise biopsies
Source: PeerJ. 2022 Feb 4;10:e12856. doi: 10.7717/peerj.12856 (PMC8820226; doi:10.7717/peerj.12856)
Supplement: Supplemental Information 5 [file peerj-10-12856-s005.docx]

S5 Table: mRNA content at baseline of all gene isoforms.

| Gene | mRNA content at baseline (a.u.) | Biopsy time with peak mRNA content (h) |
| --- | --- | --- |
| PGC-1α4 | 0.40 | 3 |
| PGC-1α | 11.12 | 3 |
| PPARα | 2.99 | 3 |
| PPARγ | 0.02 | 48 |
| CD36 | 26.99 | 24 |
| CPT1A | 0.34 | 3 |
| PDK4 | 7.34 | 9 |
| UCP3 | 2.28 | 24 |
| TFEB | 0.15 | 24 |
| p53 | 0.13 | 48 |
| GLUT4 | 6.74 | 72 |
| NRF1 | 0.06 | 24 |
